# Structural and Functional Analysis of Viral siRNAs

**DOI:** 10.1371/journal.ppat.1000838

**Published:** 2010-04-01

**Authors:** Gyorgy Szittya, Simon Moxon, Vitantonio Pantaleo, Gabor Toth, Rachel L. Rusholme Pilcher, Vincent Moulton, Jozsef Burgyan, Tamas Dalmay

**Affiliations:** 1 School of Biological Sciences, University of East Anglia, Norwich, United Kingdom; 2 School of Computing Sciences, University of East Anglia, Norwich, United Kingdom; 3 Istituto di Virologia Vegetale, CNR, Torino, Italy; 4 Plant Virology, Agricultural Biotechnology Center, Gödöllö, Hungary; University of California San Francisco, United States of America

## Abstract

A large amount of short interfering RNA (vsiRNA) is generated from plant viruses during infection, but the function, structure and biogenesis of these is not understood. We profiled vsiRNAs using two different high-throughput sequencing platforms and also developed a hybridisation based array approach. The profiles obtained through the Solexa platform and by hybridisation were very similar to each other but different from the 454 profile. Both deep sequencing techniques revealed a strong bias in vsiRNAs for the positive strand of the virus and identified regions on the viral genome that produced vsiRNA in much higher abundance than other regions. The hybridisation approach also showed that the position of highly abundant vsiRNAs was the same in different plant species and in the absence of RDR6. We used the Terminator 5′-Phosphate-Dependent Exonuclease to study the 5′ end of vsiRNAs and showed that a perfect control duplex was not digested by the enzyme without denaturation and that the efficiency of the Terminator was strongly affected by the concentration of the substrate. We found that most vsiRNAs have 5′ monophosphates, which was also confirmed by profiling short RNA libraries following either direct ligation of adapters to the 5′ end of short RNAs or after replacing any potential 5′ ends with monophosphates. The Terminator experiments also showed that vsiRNAs were not perfect duplexes. Using a sensor construct we also found that regions from the viral genome that were complementary to non-abundant vsiRNAs were targeted *in planta* just as efficiently as regions recognised by abundant vsiRNAs. Different high-throughput sequencing techniques have different reproducible sequence bias and generate different profiles of short RNAs. The Terminator exonuclease does not process double stranded RNA, and because short RNAs can quickly re-anneal at high concentration, this assay can be misleading if the substrate is not denatured and not analysed in a dilution series. The sequence profiles and Terminator digests suggest that CymRSV siRNAs are produced from the structured positive strand rather than from perfect double stranded RNA or by RNA dependent RNA polymerase.

## Author Summary

Viral RNA is processed into short RNAs in plants, which guide a complex to the viral RNA and cause cleavage of the viral RNA. We profiled *Cymbidium ringspot virus* (CymRSV) derived short RNAs using three different methods. Profiling of viral short interfering RNAs revealed a different sequence bias for the 454 and Solexa high-throughput sequencing platforms. We also found that viral short RNAs are primarily produced from the positive strand of the virus and produced with very different frequency along the viral genome. The hybridisation approach showed that the profile of viral short RNAs is determined by the virus itself because the profiles were the same in different species and it also showed that the process was RDR6 independent. We used the Terminator exonuclease to study the 5′ end of viral short RNAs and discovered that this enzyme cannot digest double stranded RNA. A control perfect duplex was only partially processed even after denaturation. Since double stranded short RNAs can quickly re-anneal, this assay must be carried out using different concentrations of the substrate. We found that most of the CymRSV short RNAs had 5′ monophosphate and were not perfect duplexes. Taken together, these results suggest that CymRSV short RNAs are produced from the structured positive strand rather than from perfect double stranded RNA or by RNA dependent RNA polymerase. We also found that regions from the viral genome that are not complementary to highly abundant viral short RNAs were targeted in the plant just as efficiently as regions recognised by abundant short RNAs.

## Introduction

The RNA silencing based antiviral plant response is one of the best studied antiviral strategies in plants. The key element of RNA silencing based antiviral strategies is the virus derived small interfering RNA (vsiRNA), which guides the RNA induced silencing complex (RISC) to target viral genomes in plants and invertebrates [1]. siRNAs are processed from double-stranded RNAs (dsRNA) or structured single-stranded RNAs (ssRNAs) by RNase III-like enzymes such as DICER [2],[3] (in plants there are several Dicer-like (DCL) genes). siRNAs guide the sequence-specific inactivation of target mRNAs by RISC [4].

Plant RNA viruses are strong inducers as well as targets of RNA silencing and high levels of vsiRNAs accumulate during the viral infection. However, despite of the extensive studies of siRNA biogenesis the origin of plant viral siRNA is still not understood. vsiRNAs are thought to be processed from ds viral RNA replication intermediates, local self-complementary ds regions of the viral genome or through the action of RNA-dependent RNA polymerases (RDRs) on viral RNA templates [1]. In plants two distinct classes of vsiRNAs have been identified: the primary siRNAs, which result from DCL mediated cleavage of an initial trigger RNA, and secondary siRNAs, whose biogenesis requires an RDR enzyme [5],[6].

DCL4 and DCL2 are the most important plant DICERs involved in virus induced RNA silencing and they can process ds or hairpin viral RNAs into vsiRNAs of 21 and 22 nt, respectively. Although DCL4 is the major player in vsiRNA production, in the absence of DCL4, DCL2 is also sufficient to produce 22 nt vsiRNA, which are biologically active in antiviral silencing response [7],[8]. siRNAs are associated with distinct Argonaute (AGO)-containing effector complexes to guide them to their RNA target molecules [1],[9],[10]. In plants, loading of siRNAs into a particular AGO complex is preferentially, but not exclusively, dictated by their 5′ terminal nucleotides [11]. AGO1 is the major slicer in plants but other AGO paralogs are likely to be involved, potentially also mediating translational repression [1],[11].

The accumulation of vsiRNAs may also depend on the presence of virus expressed silencing suppressor proteins. Several silencing suppressor proteins sequester the primary vsiRNAs thus inhibiting the accumulation of secondary vsiRNAs and the antiviral response [1],[12].

In *Arabidopsis*, RDR6 and RDR2 are required for virus (but not for all viruses) or sense transgene triggered RNA silencing, the spread of long-range cell-to-cell silencing, and the reception of the long-distance RNA silencing signal [1],[13],[14],[15]. RDR-dependent biogenesis of vsiRNAs was first demonstrated by Diaz-Pendon et al. [16]. Recently it was also shown that the production of *Tobacco rattle virus* (TRV) derived vsiRNAs and antiviral silencing are strongly dependent on the combined activity of the host-encoded RNA-dependent RNA polymerases such as RDR1, RDR2, and RDR6 suggesting that viral single-stranded RNAs, might be converted by RDR enzymes to dsRNAs, which could serve as a substrate for vsiRNA production [17]. However, this model is not supported by previous observations that the majority of vsiRNAs are derived from the plus (mRNA sense) viral strand [18],[19],[20]. In addition, it has been also shown that RDR6 is not required for virus-induced gene silencing when the endogenous phytoene desaturase (PDS) gene was silenced using the crucifer strain of tobacco mosaic virus (crTMV) and TRV based vectors [13]. These conflicting observations indicate that our knowledge of vsiRNA biogenesis is still far from complete.

*Cymbidium ringspot virus* (CymRSV) is a member of the *Tombusvirus* genus containing a positive single-stranded RNA genome with five open reading frames (ORFs) [21]. It is widely assumed that positive-strand RNA viruses replicate their genomes via dsRNA intermediates that may activate the siRNA generating machinery [22]. However, the accessibility of long viral dsRNA intermediates for DICER cleavage have not been proven experimentally. In addition, our previous studies suggested that highly structured viral RNAs might also be processed into vsiRNAs in virus-infected plants [18],[19],[23]. We have also shown that sensor RNAs with negative viral polarity are better targets of RISC mediated cleavage in CymRSV infected plants than those with positive polarity, which further suggests an excess of biologically active vsiRNAs with positive polarity [24].

In this work we analysed the composition and the molecular nature of vsiRNAs in virus infected plants in order to gain a better insight into the biogenesis of vsiRNAs using the high throughput 454 and Solexa sequencing strategies.

## Results

### Profile of viral short RNAs in CymRSV infected plants

To establish the profile of viral siRNAs, cDNA libraries of short RNAs were generated using total RNA extracted from the first systemically infected leaves of *Nicotiana benthamiana* infected with *in vitro* transcripts of either wild type CymRSV or with a mutant form of the virus that did not express the p19 silencing suppressor protein (Cym19stop). The cDNA libraries were sequenced on the 454 platform yielding around 100 000 sequences for each library (Table 1). The short RNA sequences were mapped to the viral genome and we found 65% of the reads on the positive strand of wild type CymRSV without mismatches. The mutant virus also produced vsiRNAs at a similar ratio from the positive strand (63.5%) indicating that the silencing suppressor did not affect strand preference and that the higher representation of plus strand is consistent. Next we assessed whether deep sequencing also identifies vsiRNA hot spots as small scale vsiRNA sequencing did [19]. The abundance profiles of vsiRNAs on the positive and negative strands of viral genomic RNA were very similar for wild type and mutant viruses (Figure S1A and B): vsiRNAs were produced from the entire genome with several hot spots on the positive strand and a few low abundance hot spots on the negative strand. The position of the hot spots were identical between wild type and mutant viruses indicating that p19 does not influence the generation of high abundance vsiRNAs and that the 454 platform yielded reproducible vsiRNA profiles.

**Table 1:** Summary of 454 sequencing.

	CymRSV	Cym19stop
Total reads	113587	91393
Adaptors removed	88421	73257
Matching viral genome (0 or 1 mismatch)	73252	43741
Positive strand	47807 (65.26%)	28636 (65.47%)
Negative strand	25445 (34.74%)	20292 (46.39%)
Matching viral genome (0 mismatches)	57453	31907
Positive strand	37221 (64.79%)	20292 (63.60%)
Negative strand	20232 (35.21%)	11615 (36.40%)
Total non-redundant sequences	34136	38293
Non-redundant sequences mapping to viral genome (0 or 1 mismatch)	14892	13008
Positive strand	8265 (55.5%)	7031 (54.05%)
Negative strand	6627 (44.5%)	5977 (45.95%)
Non-redundant sequences mapping to viral genome (0 mismatches)	11835	6949
Positive strand	6419 (54.23%)	3663 (52.71%)
Negative strand	5416 (45.76%)	3286 (47.29%)

**Figure 1:**
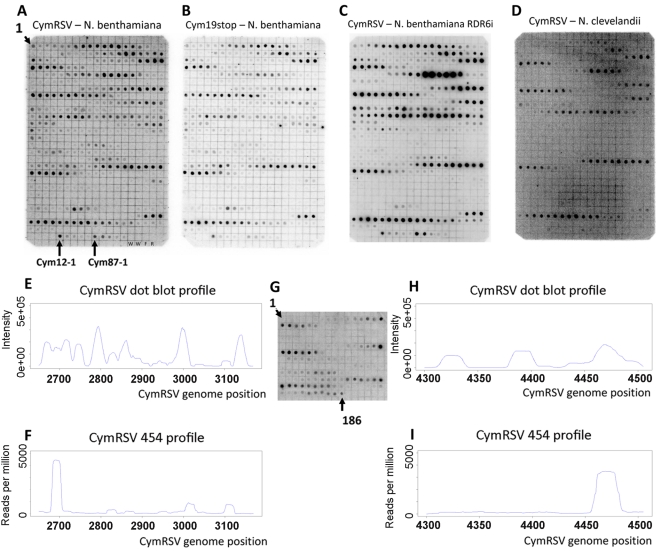
Dot blot and 454 profile of vsiRNAs. Short RNA fraction was purified from *in vitro* transcribed virus infected plants, dephosphorylated, 5′ labelled and hybridised to membranes containing 21mer oligonucleotides in a one nucleotide sliding window complementary to the plus strand of CymRSV in positions 2650–3169 (A–D) or 4300–4505 (G). Tissues of wild type *N. benthamiana* infected with either wild type CymRSV (A and G) or with p19 deficient mutant CymRSV (B) and either transgenic *N. benthamiana* with reduced RDR6 expression (C) or wild type *N. clevelandii* (D) infected with wild type CymRSV were used for RNA extraction. Signal intensity of dots on panel A was quantified and plotted along the viral genome (E) and compared with the read frequency for the same region obtained through 454 sequencing (F). Signal intensity of dots on panel G was quantified and plotted along the viral genome (H) and compared with the read frequency for the same region obtained through 454 sequencing (I). The layout of the membranes in panel A–D is the same since the same membrane was used after stripping. The position of the most 5′ oligonucleotides is indicated as 1 at the top left corner. The membranes contain two kinds of negative controls: two dots contain only water (W) and two non-specific oligonucleotides are M13 forward (F) and reverse (R). We monitored the specificity of hybridisation by dotting shorter oligonucleotides: an abundant probe (Cym12) was shortened from the 5′ end by 1, 2, 3, 4 and 5 nucleotides (Cym12-1 indicates the 20mer probe followed by the shorter probes). The same approach is used for another abundant probe Cym87.

Next we wanted to experimentally validate the positions of the hot spots obtained by 454 sequencing. A 520 nt region (nucleotides 2650–3169 on the positive strand) containing one highly abundant and a few less abundant hot spots on the positive strand was selected for validation. Five hundred 21mer oligonucleotides (complementary to the plus strand of CymRSV) in a one nucleotide sliding window were designed to cover the entire selected region and spotted on a membrane. The short RNA fractions (19–24 nt) were isolated from first systemic leaves of plants infected with *in vitro* transcripts of wild type and mutant virus, labelled at the 5′ end and used as probes to hybridise to the membrane. The two dot blots gave very similar if not identical patterns (Figure 1A and B) providing experimental evidence that hot spots do exist and p19 does not influence the position of hot spots. The membranes were also hybridised to labelled short RNAs isolated from wild type CymRSV *in vitro* transcript infected *Nicotiana clevelandii* and a *N. benthamiana* where the RDR6 gene [13],[14] was silenced by an inverted repeat construct [15] (Figure 1C and D). These probes gave identical patterns to the previous two probes suggesting several conclusions. First, it indicated that viral siRNAs were generated from hot spots in the absence of RDR6 therefore this RNA dependent RNA polymerase was not absolutely essential for the increased production of CymRSV vsiRNAs from certain positions of the viral genome. Second, it showed that the positions of hot spots only depend on the viral genome and are not determined by the host since the hot spots mapped to the same regions in two different hosts. The fact that four experiments gave identical patterns of hot spots proved that the experimental approach is reproducible. Intensity of the dots was quantified and plotted along the selected region to obtain vsiRNA profiles. Comparison of vsiRNA profiles obtained by 454 and hybridisation, however, showed no correlation (Figure 1E and F), although both approaches were reproducible. We carried out another dot blot experiment covering a different region on the viral genome (4300–4505) and obtained similar result (Figure 1G, H and I). One of the differences between the two methods is that the short RNAs were de-phosphorylated before labelling the 5′ end while the 5′ 454 adapter was ligated directly without de-phosphorylation. If many short RNAs contain a 5′ end that cannot ligate directly to an adapter but can be labelled after de-phosphorylation that could explain the difference between the two sets of results. Alternatively, the two approaches have different preferences in detecting vsiRNA sequences. To distinguish between these possibilities first we analysed the 5′ end structure of vsiRNAs using the Terminator™ 5′-Phosphate-Dependent Exonuclease and then we profiled vsiRNAs using a different high-throughput sequencing platform after ligating the 5′ adapter either directly or following de- and re-phosphorylation to short RNAs extracted from plants inoculated with *in vitro* transcripts of either wild type or Cym19stop viruses.

### Analysis of the 5′ end of viral siRNAs

**Figure 2:**
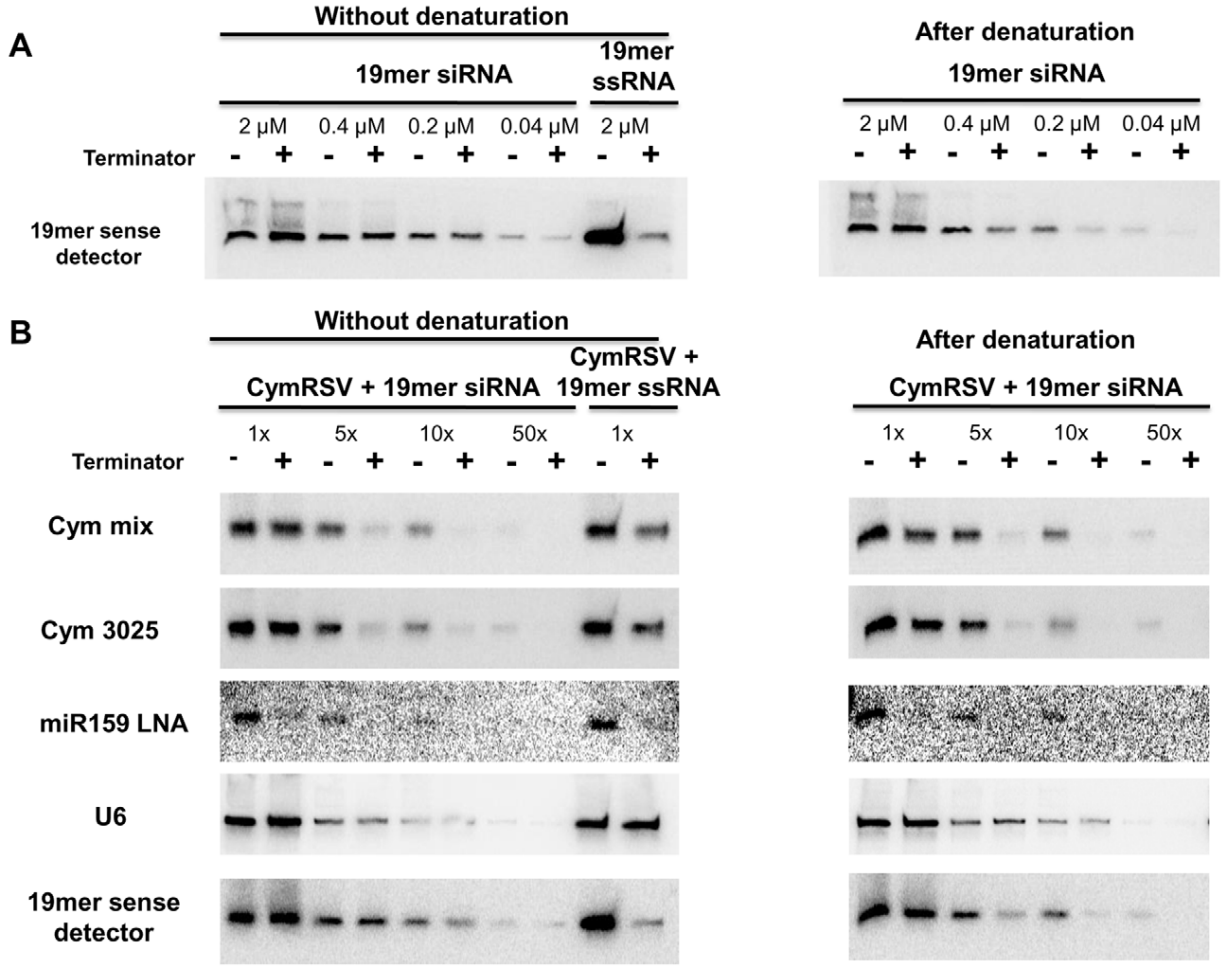
Terminator digest of synthetic siRNAs and vsiRNAs. A 19mer *in vitro* synthesised and phosphorylated siRNA was digested with Terminator™ 5′-Phosphate-Dependent Exonuclease in decreasing concentration without (left panel) or after denaturation (right panel)(A). The sense strand of the phosphorylated 19mer RNA oligonucleotide was digested in the highest concentration as a control for phosphorylation. The efficiency of the digestion was monitored by northern blot assay using a probe complementary to the sense strand of the synthetic siRNA. Total RNA from CymRSV infected *N. benthamiana* plants was mixed with the 19mer *in vitro* synthesised and phosphorylated siRNA and digested with Terminator™ 5′-Phosphate-Dependent Exonuclease in decreasing concentration without (left panel) or after denaturation (right panel) (B). The sense strand of the phosphorylated 19mer RNA was also mixed with total RNA from CymRSV infected *N. benthamiana* plants and digested in the highest concentration. The efficiency of the digestion was monitored by northern blot assay using probes complementary to the minus strand of the virus (Cym mix, a pool of five probes or Cym 3025), miR159, U6 snRNA or the sense strand of synthetic siRNA, respectively.

To decide whether a substantial percentage of vsiRNAs cannot be ligated directly to an adapter through the 5′ end we used the terminator exonuclease to study the 5′ end of vsiRNAs. This enzyme degrades RNA molecules in a 5′ to 3′ direction but it can only process molecules with a monophosphate at the 5′ end [25], however it is not known whether short dsRNA molecules can be processed by this enzyme. It was important to address this question since vsiRNAs are double stranded [19] therefore we digested a perfect duplex (with two nucleotide overhangs at the 3′ ends), which consisted of two 19mer *in vitro* synthesised and phosphorylated RNA oligonucleotides in decreasing concentration without or after denaturation and compared these with undigested samples (Figure 2A). The enzyme was not able to digest the non-denatured perfect duplex at all (Figure 2A left panel) indicating that these molecules are not substrates of this enzyme even if they have 5′ monophosphate end. Most of the RNA was digested by the exonuclease when only one of the strands from the duplex was digested, which demonstrates that the enzyme was functional and that the RNA oligonucleotides were efficiently phosphorylated. The enzyme was able to partially digest the duplex RNA if it was denatured before incubating with the enzyme (Figure 2A right panel). This experiment was repeated using two other perfect duplexes and the same result was obtained (Figure S3B).

RNA extracted from CymRSV *in vitro* transcript infected plants was treated with terminator exonuclease without or after denaturation and the level of different short RNAs was compared with an untreated sample by northern blotting. We used a miRNA probe as a positive control for the treatment because it is known that the 5′ end of miRNAs contain a monophosphate and so they are expected to be processed by terminator exonuclease. We also added the 19mer perfect duplex to the samples as an additional control. The miRNAs were indeed efficiently degraded by the terminator (Figure 2B miR159 probe) providing an internal control for the enzyme reaction. The perfect duplex gave the same result as previously: it was not digested at all without denaturation and was partially digested after denaturation (Figure 2B 19mer probe). Two different probes were used to analyse the viral siRNAs (Figure 2B Cym probes) and they gave identical results: vsiRNAs were partially digested with or without denaturation and the efficiency of the digestion was increased dramatically by diluting the sample (two more viral probes are shown on Figure S2). Comparing the digestion efficiency of the vsiRNAs and the perfect duplex after denaturation we can conclude that the vsiRNAs are even more efficiently digested than the 19mer duplex. This indicates that most vsiRNAs must have monophosphate at the 5′ end. The weak signal in the digested lanes of the diluted samples could be either due to renaturation or a small amount of 5′ triphosphate siRNAs. Based on this experiment we cannot rule out that a small amount of vsiRNAs have 5′ triphosphate but we can conclude that the majority of CymRSV vsiRNA possess 5′ monophosphate.

**Figure 3:**
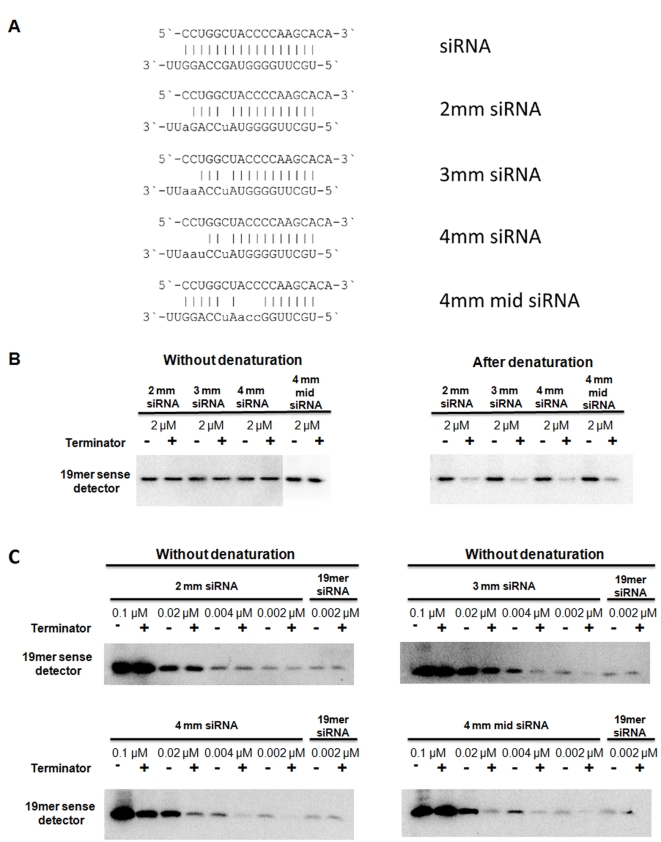
Terminator digest of imperfect duplexes. *In vitro* synthesised and phosphorylated imperfect RNA duplexes (A) were digested with Terminator™ 5′-Phosphate-Dependent Exonuclease at the concentration of 2 µM without (left panel) or after denaturation (right panel) (B). The efficiency of the digestion was monitored by northern blot assay using a probe complementary to the sense strand of the imperfect duplexes, which was identical in all duplexes. 2mm, 3mm and 4mm siRNAs contained 2, 3 or 4 mismatches, respectively. 4mm mid siRNA also contained 4 mismatches but at a more central location. The imperfect duplexes were digested at a decreasing concentration without denaturation (C). As a control, the perfect duplex was digested at the lowest concentration (0.002 µM).

We also compared the digestion efficiency of vsiRNAs and the control perfect duplex without denaturation and found that vsiRNAs were much more efficiently digested without denaturation than the perfect duplex. This raises the possibility that the vsiRNAs are not perfect duplexes but imperfect dsRNA molecules with mismatches between the two strands as it was proposed previously [19]. To explore this we used synthetic imperfect duplexes with 2, 3 or 4 mismatches between the strands (Figure 3A and Figure S3C). Two kinds of duplexes were used with 4 mismatches that differed in the position of the mismatches (Figure 3A). First we digested all four imperfect duplexes at 2 µM concentration either without or after denaturation (Figure 3B). None of the imperfect duplexes were digested without denaturation but all of them were efficiently digested after denaturation. This showed that imperfect duplexes are digested more efficiently after denaturation than perfect duplexes because the perfect duplex was not digested at all at this concentration even after denaturation (compare right panels of Figure 2A and Figure 3B). Next we compared the digestion pattern of imperfect duplexes and vsiRNAs without denaturation by diluting each of the four imperfect duplexes and digesting them without denaturation (Figure 3C). The perfect duplex was used as a control and it was not digested even at the lowest concentration we tried (0.002 µM). The imperfect duplex with two mismatches showed a very small amount (if any) digestion at 0.002 µM but not at higher concentrations. The imperfect duplex with three mismatches was digested much more efficiently, showing big differences between digested and non-digested samples at concentrations 0.002 µM and even 0.004 µM. The imperfect duplex with four mismatches at the end of the duplex showed the most efficient digestion, detected even at 0.1 µM, while the same number of mismatches but at the middle of the duplex resulted efficient digestion at a slightly lower concentration (0.02 µM). Two more imperfect duplexes showed similar pattern (Figure S3C). This set of experiments demonstrated that perfect and imperfect duplexes show different digestion pattern by terminator exonuclease without denaturation and that vsiRNAs showed a similar pattern to the imperfect duplexes: they are both digested at the “right concentration”. The right concentration depends on the number and position of mismatches.

### Profiling vsiRNAs in CymRSV infected plants by Solexa sequencing

We also studied the 5′ end of vsiRNAs by ligating another adapter molecule (different from the 454 5′ adapter) to the 5′ end of vsiRNAs. cDNA libraries of short RNAs were generated using total RNA extracted from early systemic leaves of *N. benthamiana* infected with *in vitro* transcripts of either wild type CymRSV or with a mutant virus, Cym19stop. The library generation includes an adaptor ligation to the 5′ end of the short RNAs and this can be done either after removing the phosphate(s) from the 5′ end or without modifying the 5′ end. When short RNAs are dephosphorylated the 5′ monophosphate, diphosphate or triphosphate is removed and then the short RNAs are re-phosphorylated ensuring that all short RNAs have a 5′ monophosphate.

Solexa sequencing of the four libraries resulted in over one and a half million reads for each library (Table 2). The ratio of vsiRNAs mapping to the positive and negative strands was identical for the wild type virus infected plants when the short RNAs were directly ligated to the adapter or after de- and re-phosphorylation. The high percentage of vsiRNAs derived from the positive strand (93%) confirmed once more that most vsiRNAs are not processed from the double stranded (ds) replicative form. This result also shows that the bias for positive strand sequences is not due to a modification at the 5′ end of the vsiRNAs because the two ligation approaches gave identical ratios. The ratio of positive/negative strand vsiRNAs and their distribution was also very similar in the libraries obtained by the two different ligation methods using RNA extracted from the mutant virus (Cym19stop) infected plants. However, the percentage of vsiRNAs generated from the negative strand of the mutant virus (Cym19stop) was higher than it was for the wild type virus (22–29% compared to 5–6%; Table 2). We hypothesised that this increase was due to higher ratio of negative strand genomic RNA to positive strand genomic RNA in the mutant virus infected plants compared to wild type virus infected plants and this was confirmed by Northern blot analysis (Figure S4).

**Table 2:** Summary of Solexa sequencing.

	CymRSV-PHOS	CymRSV-CIP	Cym19stop-PHOS	Cym19stop-CIP
Total reads	1814303	4680503	4224425	2725324
Adaptors removed	1642450	4127290	3147200	2472857
Matching viral genome (0 or 1 mismatch)	1291275	3164592	2486423	1068015
Positive strand	1209070 (93.6%)	2968299 (93.8%)	1742090 (70.1%)	818437 (76.6%)
Negative strand	82205 (6.4%)	196293 (6.2%)	744333 (29.9%)	249578 (23.4%)
Matching viral genome (0 mismatch)	1176671	2907947	2078336	935598
Positive strand	1107492 (94.12%)	2741730 (94.28%)	1471956 (70.82%)	722927 (77.27%)
Negative strand	69179 (5.88%)	166217 (5.72%)	606380 (29.18%)	212671 (22.73%)
Total non-redundant sequences	211564	334003	320540	339965
Non-redundant sequences mapping to viral genome (0 or 1 mismatch)	73905	104993	107468	70004
Positive strand	58589 (79.3%)	83662 (79.7%)	64273 (59.8%)	43173 (61.7%)
Negative strand	15316 (20.7%)	21331 (20.3%)	43195 (40.2%)	26831 (38.3%)
Non-redundant sequences mapping to viral genome (0 mismatch)	28833	35380	29303	30094
Positive strand	19857 (68.9%)	23569 (66.6%)	16111 (55.0%)	16976 (56.4%)
Negative strand	8976 (31.1%)	11811 (33.4%)	13192 (45.0%)	13118 (43.6%)

Although most redundant vsiRNAs matched perfectly the viral genome, we observed a higher ratio of not perfectly matching non-redundant reads/perfectly matching non-redundant reads for the positive strand vsiRNAs compared with the negative strand vsiRNAs (p<0.05). One possible explanation for this is the fact that a lot more sequences are derived from the positive strand therefore there is a higher probability to observe sequencing errors in positive strand vsiRNAs. In fact the difference is more pronounced for the wild type virus compared to the mutant virus most likely because the positive/negative strand vsiRNA ratio is higher for the wild type (15×) than for the mutant (2.7×) sample. We also simulated the effect of sequencing errors on positive and negative strand vsiRNAs. The perfectly matching positive and negative strand vsiRNAs were separated from the wild type data set and different rates of sequencing errors (1 error in 1000, 100 or 10 sequences) were simulated. The ratio of perfectly matching sequences to those matching with 0 or 1 mismatch was calculated. We found a similar effect on the positive and negative strand vsiRNAs when redundant reads were used but a much more pronounced effect on the positive strand vsiRNAs when the simulation was applied to non-redundant reads (Figure S5). This result confirms that the observed difference in ratios of not perfectly matching non-redundant reads/perfectly matching non-redundant reads for the positive strand vsiRNAs compared with the negative strand vsiRNAs can be due to sequencing errors. The other contributing factor could be if the not perfectly matching reads are not sequencing errors but vsiRNAs derived from mutated viral genomes. Positive strands can contain point mutations that are not present on the negative strand if no negative strand is produced from the mutant positive strand. However, it is less likely that the negative strand contains a mutation that is not present on the positive strand since the positive strands are produced from the negative strands.

**Figure 4:**
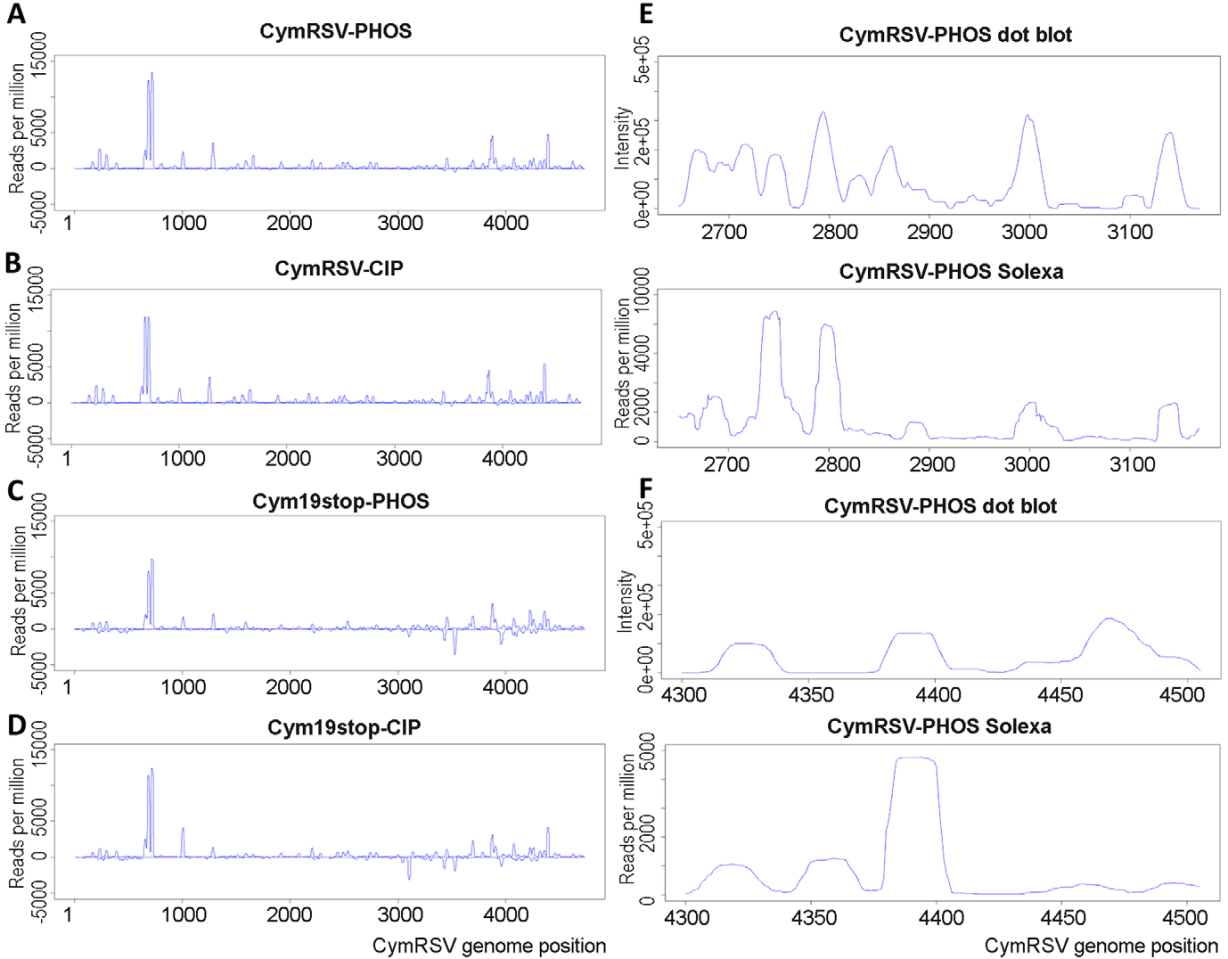
Solexa and dot plot profiles of vsiRNAs. vsiRNA sequences obtained by Solexa sequencing were mapped to the CymRSV genome and normalised abundances were plotted for each sample (A–D). Short RNAs were sequenced from wild type (CymRSV) or silencing protein disabled (Cym19stop) *in vitro* transcript infected *N. benthamiana* following two different protocols. The 5′ adapter was either directly ligated to the short RNAs (PHOS) or first depohosphorylated and then re-phosphorylated before adapter ligation (CIP). Profiles obtained by dot blot and Solexa sequencing were compared for regions 2650–3169 (E) and 4300–4505 (F). Please note that the dot blot profiles are the same as those shown in Figure 1E and H.

The abundances of reads in all four libraries were plotted on the viral genome (Figure 4A, B, C and D) and hot spots were located. Comparison of the vsiRNA profiles obtained by Solexa and the dot blot approaches revealed a high similarity between the two profiles (Figure 4E and F). Since the Solexa platform gave more similar result to the dot blot than the 454 we analysed the Solexa profile further (direct comparison of the vsiRNA profiles obtained by 454 and solexa is shown on Figure S6).

Size distribution and 5′ nucleotide bias was compared between the four libraries and specifically between vsiRNAs derived from hot spots (Figure S7). A slight bias was found for A and U at the 5′ position, especially in wild type virus infected plants but regardless of the ligation method. We also found a slight G bias in the first position of 22mer reads in the libraries from plants infected with the mutant virus. This bias was slightly more pronounced in the vsiRNAs derived from hot spots but there was no other significant difference in the size distribution and 5′ nucleotide bias between the total population of siRNAs and siRNAs generated from hot spot regions. The main difference revealed by this analysis was that the most abundant class of siRNAs in plants inoculated with wild type virus was the 21 nt sequences followed by the 22 nt class while in plants inoculated with the mutant virus the 22 nt siRNAs were more abundant than the class of 21 nt (the same was found by 454 sequencing; data not shown). To further investigate this difference we plotted the 21 and 22 nt sequences separately for all four libraries (Figure S8). The plots show a very similar pattern for 21 and 22 nt sequences in all four libraries and a very similar high positive/negative strand ratio.

### Identifying potential vsiRNA duplexes

All of our results (including the presence of hot spots, the + strand bias, the independence of hot spots from RDR6, the similar pattern of libraries generated by direct ligation or after dephosphorylation and the similar profile of wild type and mutant viruses; please note that p19 blocks the generation of primer dependent secondary siRNAs) suggest that vsiRNAs are produced from structured + or − strands, rather than from the double-stranded replicative form or from host RDR generated double-stranded RNA. To explore this further one could predict the secondary structure of the viral genomic RNA or the hot spot regions. However, prediction of RNA secondary structure of long molecules is not reliable. It is also very likely that long RNA molecules have several possible conformations, which is difficult to model by using computational methods. Prediction of shorter regions is more reliable but this approach completely ignores that sequences far away from each other can pair with each other. Therefore secondary structures of hot spot regions are not informative either. We took a different approach and investigated whether the sequence reads derived from the + strand can potentially form duplexes with each other allowing up to four mismatches. We found a large number (8492) of such potential duplexes (Table S1) suggesting that vsiRNA duplexes indeed can be derived from the positive strand only. A similar approach identified 3617 imperfect duplexes that can derive from the − strand (Table S2).

### Functional study of vsiRNAs

**Figure 5:**
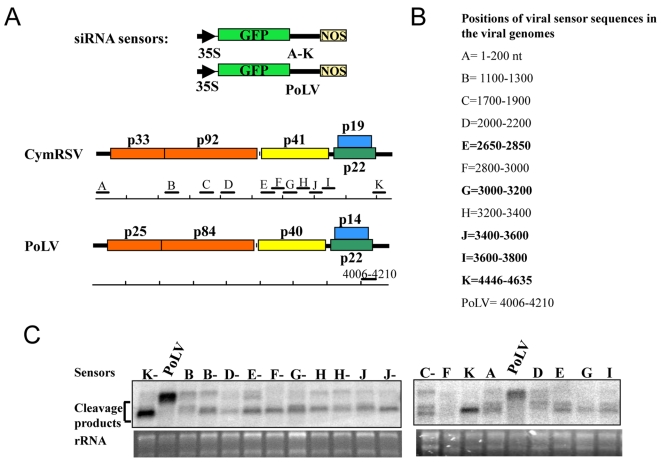
Functional analysis of vsiRNAs *in planta*. A: Schematic illustration of the GFP sensor sequences and their origins in the viral genomes. The GFP and viral coding sequences are indicated by coloured open boxes, and non coding regions are indicated by thick black lines. Viral target sequences, CymRSV: A to K and Photos Latent Virus (PoLV) [24] as a control, inserted downstream to the GFP ORF are scaled and positioned along the viral genomes. B: Length and position of viral sensor sequences are listed, and regions that contain hotspots are shown in bold. C: Northern blot analysis of viral sensor mRNAs agroinfiltrated into Cym19stop-infected N. benthamiana plants. RNA extracts at 3 days after agroinfiltration were analysed with a ^32^P-labeled RNA probe raised against the 3′-terminal part of the GFP ORF. PoLV indicates the negative uncleaved control, while the CymRSV viral sequences are indicated by the capital letters. The letter followed by “-” indicates the negative orientation of the sensor sequence, rRNA was used as loading control.

In order to understand the biological role of hot spots, we carried out a functional analysis of viral siRNAs. Different regions (200 nt length) of the viral genome in both plus and minus orientation were selected from hot spots and from non hotspots regions (Figure 5). These were cloned downstream of a green fluorescent protein (GFP) reporter gene and expressed from a binary vector. The reporter constructs were agroinfiltrated into systemically infected leaves of *N. benthamiana* plants inoculated with *in vitro* transcript of Cym19stop virus. In the absence of the silencing suppressor protein the viral siRNAs are incorporated into RISC and target the GFP mRNA if they are complementary to the viral fragment in the sensor construct [24],[26]. Some constructs contained fragments including hot spots and other constructs carried fragments that did not include hot spots. All sensor constructs were targeted by vsiRNAs, although with variable efficiency and without clear correlation with vsiRNA hotspots (Figure 5). In fact, some fragments not homologous to any hot spot (Figure 5, lanes F-) showed similar down-regulation to constructs with hot spot regions (Figure 5, lanes G-). This result indicates that vsiRNAs generated from hot spots are not more efficient than other vsiRNAs in spite of their much higher abundance.

## Discussion

### vsiRNA profiles in CymRSV infected plants

We used three approaches to profile vsiRNAs: 454 and Solexa high-throughput sequencing and hybridisation of 5′ labelled vsiRNAs to two arrays of DNA oligonucleotides covering a 520 and a 206 nt region of the viral genome. All three approaches gave reproducible results because total RNA extracted from wild type or suppressor deficient CymRSV infected plants gave very similar results for each technique. The hybridisation and Solexa sequencing showed good correlation with each other whereas the profile obtained using 454 sequencing was not in agreement. This suggests that ligation of the 454 adaptors have some sequence preference and not necessarily the most abundant molecules are present at high frequency in the cDNA library. This was also reflected by a bias to sequence reads starting with C nucleotide in the 454 library (data not shown) compared with a bias for U and A in the Solexa library (Figure S7). This however, does not mean that 454 is not appropriate to profile short or mRNAs in different samples. The 454 profile was reproducible demonstrating that the preference shown for certain sequences is the same in different samples therefore it is still a valid technique to compare the abundance of transcripts/sRNAs between samples. During revision of this manuscript it was reported that different high-throughput sequencing platforms and different library construction protocols led to different profiles within the same sample [27]. The reason for these differences is not known but is probably influenced by the sequence of the short RNAs to be profiled. Because of this it is possible that different platforms/protocols would give a more accurate profile in different systems. Clearly more studies are necessary that use different platforms in the same system, and in different systems in order to obtain a comprehensive picture about the reliability of the different platforms.

Both sequencing approaches identified more negative strand vsiRNAs in the p19 mutant virus infected plants, which mainly mapped to the last 1700 nt of the viral genome (downstream of nucleotide 3000). This shows a good correlation with the strongly increased level of subgenomic RNA 2 negative strand in the mutant virus infected plants compared with wild type virus infected plants, suggesting that the subgenomic RNAs also contribute to the vsiRNA pool.

The Solexa and array approaches both identified regions from which vsiRNAs were generated at a higher than average frequency. Although this was reported previously based on small scale sequencing of vsiRNAs it could not be ruled out that those observations were influenced by the small data size. This comprehensive study confirmed that there are hot spots on the viral genome that produce specific vsiRNAs at a very high abundance. We also showed that the positions of these hot spots are the same in at least two different hosts indicating that it is determined by the virus itself. The profile of vsiRNAs was also identical in wild type plants and in plants where RDR6 accumulation was suppressed. The symptoms of these two plants following CymRSV infection were also very similar (data not shown) demonstrating that RDR6 is not required for vsiRNA production from the CymRSV genome. Some viruses, such as cucumber mosaic virus [14] and potato virus X [15] cause more severe symptoms in the absence of RDR6 than on wild type plants but many other viruses including tobacco rattle virus, tobacco mosaic virus, turnip crinkle virus cause similar symptoms in the presence and absence of RDR6 [13]. This indicates that production of siRNAs from some viruses requires RDR6 but this enzyme is not involved in vsiRNA production from other viruses, including CymRSV. Since there are several other RDR family members in plants it is possible that one or more other RDRs are involved in CymRSV vsiRNA production through two potential mechanisms. For example it was reported recently that RDR1 is involved in vsiRNA production in TMV-Cg infected plants [28]. The first mechanism is similar to the generation of DCL-dependent (DCL-D) secondary siRNA production from transgenes [1] where primary siRNAs prime the synthesis of complementary RNA of the silenced RNA by RDR. The resulting dsRNA is processed by one of the DCL genes producing DCL-D secondary siRNAs. These DCL-D secondary siRNAs contain 5′ monophosphate like primary siRNAs that are also produced by one of the DCLs but independently of any RDR enzyme. The second possible mechanism was described in *C. elegans* where an RDR directly produces siRNAs without Dicer activity [25] (DCL-I; DCL independent). However, our data indicate that in the case of CymRSV none of these mechanisms play an important role as discussed next.

### DCL-dependent secondary siRNAs are not abundant in CymRSV infected plants

The CymRSV encoded silencing suppressor (p19) was shown to bind primary siRNAs and suppress the accumulation of DCL-D secondary siRNAs by blocking the activity of the primary siRNAs [26]. This indicates that if DCL-D secondary siRNAs were produced at a high level in CymRSV infected plants than we should observe a different vsiRNA profile in the absence of the viral suppressor. In this scenario Cym19stop infected plants would contain more DCL-D secondary siRNAs than the wild type virus infected plant and the vsiRNA profile would be different in the two plants. However, we did not obtain different vsiRNA profiles in wild type and mutant virus infected plants indicating that most CymRSV siRNAs are primary siRNAs and not DCL-D secondary siRNAs. Moreover the vsiRNA profile of CymRSV infected RDR6 silenced plants was the same as the virus infected wild type plants further supporting our conclusion since RDR6 is involved in DCL-D secondary siRNAs biogenesis.

### Dicer-independent viral siRNAs are not abundant in CymRSV infected plants

Although DCL-I secondary siRNAs have not been described in plants, plant virus derived siRNAs are good candidates for being direct products of an RDR. Most plant viruses are RNA viruses therefore vsiRNAs could be produced by the viral RDR or alternatively by one of the host RDRs. In addition, vsiRNAs were shown to be generated from hot spots on the viral genome that could be preferred sites of unprimed RNA synthesis. We took two different experimental approaches to analyse the 5′ structure of vsiRNAs at a genomic scale because DCL-I secondary siRNAs possess 5′ triphosphates (since the 5′ ends are not generated by Dicer cleavage but by RNA synthesis) and primary siRNAs have 5′ monophosphate. First we digested RNA extracted from virus infected plants with the terminator exonuclease and compared the level of vsiRNAs in treated and untreated samples by Northern blot. A similar level of vsiRNA in treated and untreated samples would suggest that most vsiRNAs carry 5′ triphosphates since the enzyme can only process molecules with 5′ monophosphate but not molecules with 5′ triphosphates. Our experiments revealed that most of the CymRSV vsiRNAs were processed by the enzyme when the samples were diluted thus we concluded that most vsiRNAs carry a 5′ monophosphate, although it cannot be ruled out that there is a small amount of vsiRNA with 5′ triphosphates.

We also used another approach to study the 5′ structure of vsiRNAs where we profiled the short RNAs after ligating an adaptor to the 5′ end either directly or after de- and re-phosphorylation. The direct ligation only reveals vsiRNAs with 5′ monophosphate while the other approach also identifies vsiRNAs with 5′ triphosphate. If the profiles are different that suggests that there are vsiRNAs with 5′ triphosphate or diphosphate ends because they would be included in the dephosphorylated library but not in the directly ligated library. However, if the sequence profiles of the two libraries are the same that would indicate that there are not vsiRNAs with 5′ triphosphate or diphosphate because removing the 5′ phosphates does not have any effect. The patterns of vsiRNA hot spots in plants infected with the wild type virus were identical when the short RNAs were ligated to the 5′ adapter directly or after de- and re-phosphorylation. This argues against the idea that vsiRNAs in hot spots are mainly siRNAs with triphosphate 5′ end because in that case we should have seen a shift between the patterns obtained in the two experiments. The mutant virus also generated a similar vsiRNA profile regardless of the ligation method.

### Terminator exonuclease does not digest double stranded short RNA and its efficiency strongly depends on the concentration of the substrate

To investigate the 5′ end of vsiRNAs we also used another approach that is based on the ability of the Terminator™ 5′-Phosphate-Dependent Exonuclease to distinguish between 5′ monophosphate and 5′ triphosphate (i.e. only digests RNA with 5′ monophosphate). Since a negative result was possible in the terminator exonuclease experiments (i.e.: no digestion of vsiRNAs), it was important to monitor the enzyme activity. A possible internal control is to monitor the level of miRNAs before and after exonuclease treatment since this class of short RNAs has 5′ monophosphate and present in the RNA extracted from virus infected plants. However, we observed during our preliminary experiments that different samples showed different ratios of digested vsiRNAs while the miRNA was always completely digested (data not shown). We hypothesised that a big difference between vsiRNAs and miRNAs is their accumulation level. Small scale short RNA cloning and sequencing experiments found that the majority of the short RNA population in virus infected plants is virus derived [19]. Therefore we decided to use a synthetic siRNA duplex as an additional control. Another difference between miRNAs and vsiRNAs is that most miRNAs are incorporated into RISC quickly while most vsiRNAs are not co-purified with RISC [24]. As a consequence, most miRNAs are present as single stranded RNA and most vsiRNAs accumulate as dsRNA [19]. This raised the question whether the terminator exonuclease have lower affinity to dsRNA than ssRNA. We characterised the terminator exonuclease using a perfect duplex of two 19mer *in vitro* synthesised and phosphorylated RNA oligonucleotides. We found that the enzyme did not digest the non-denatured duplex at all. The duplex was partially digested following denaturation but only at lower concentration. The fact that the duplex was not digested at all at high concentration suggests that short RNAs can re-anneal very quickly but renaturation happens less frequently at lower concentrations therefore the enzyme activity increased dramatically. These results demonstrate that any RNA sample analysed by the terminator exonuclease has to be denatured and digested at different concentrations. This conclusion has an important implication beyond the plant virology field since the terminator exonuclease can be used to study the 5′ structure of RNA molecules in diverse systems, including endogenous siRNA biogenesis in animals.

### Most CymRSV vsiRNAs are not generated from perfect dsRNAs

Interestingly, vsiRNAs were digested with a similar efficiency without or after denaturation, which is different from the efficiency of the digestion of a perfect duplex and very similar to the digestion pattern of imperfect duplexes. This suggests that vsiRNAs are not perfect duplexes but there are mismatches between the two strands. The most likely explanation is that most vsiRNAs are not generated from long perfectly matched double stranded RNAs but from precursor-miRNA like imperfect intramolecular structures. This is also supported by previous observation [19] and by the very strong strand bias of sequenced vsiRNAs: 93% of sequences obtained by the Solexa profiling derived from the positive strand. Theoretically, the imperfect duplex controls could be used to determine the number of mismatches in the vsiRNAs. However, one should know the concentration of vsiRNAs in order to compare with the concentrations of the imperfect duplexes. Unfortunately, this is very difficult since the vsiRNAs are part of the total RNA, which also contains many different plant short RNAs.

Identifying the precursor-miRNA like structures is not easy on long RNA molecules. Qi et al (2009) searched for stem-loop structures around hot spots of vsiRNAs on the TMV-Cg genome but did not find these structures [28]. However, this approach ignores the fact that distant regions of a long RNA can anneal to each other. Therefore instead of looking for local secondary structures, we identified imperfect duplexes in the sequenced library that can derive from the + or − strand. Although these imperfect duplexes do not prove that vsiRNAs derive from structured single stranded viral RNA, all our results support this model over other possibilities.

### Hierarchical activity of DCL proteins on viral RNA

It was shown in TCV (turnip crinkle virus) and TRV infected Arabidopsis plants that DCL4 is the primary nuclease to produce vsiRNAs but if DCL4 is absent or suppressed DCL2 can also produce vsiRNAs [7]. Based on this we expected to find mainly 21 nt vsiRNAs produced by DCL4. In wild type virus infected plants indeed the 21 nt class was the dominant vsiRNA class followed by the 22 nt vsiRNAs. However, in the p19 mutant virus infected plants we found more 22 nt than 21 nt vsiRNAs. The first conclusion is that the viral structures are probably recognised by multiple DCLs. DCL4 predominantly produces 21 nt vsiRNAs and DCL2 mainly generates 22 nt vsiRNAs [7]. There are at least two possible explanations for the difference in the dominant size class of vsiRNAs between mutant and wild type virus infected plants. One possibility is that the 22 nt sequences are the consequence of primary vsiRNA activity, which is inhibited by p19 and therefore less 22 nt sequences are produced in wild type virus infected plants. If the 22 nt sequences are the products of primer-dependent secondary siRNA production, they would be expected to show a similar ratio of positive to negative strand since they would be produced from a perfect dsRNA consisting of the positive and negative strand of viral genome. Alternatively, 21 and 22 nt sequences are produced both in the presence and absence of p19 (i.e. independently of primary vsiRNA activity) from the same substrate RNA (i.e. mainly the positive viral RNA strand) but because p19 binds the 21 nt duplexes more strongly, these are preferentially stabilised by p19 in wild type virus infected plants. In this scenario, both 21 and 22 nt sequences would show a similar, high positive/negative strand ratio. By plotting the 21 and 22 nt sequences separately for all four solexa libraries we found that the profiles of 21 and 22 nt sequences were very similar to each other and both class displayed a high positive to negative strand ratio. This indicates that the 22 nt sequences are not the consequence of primary vsiRNA activity. In fact the plots suggest that more 22 nt sequences are generated from the viral RNA but because p19 binds to 21 nt duplexes more efficiently, the shorter duplexes are preferentially stabilised in wild type infected plants. The fact that in the absence of p19 the 22 nt sequences accumulate at a higher level than the 21 nt class raises the possibility that DCL2 is dominant over DCL4 in *N. benthamiana* at least in the case of processing CymRSV RNA. However, this hypothesis has to be tested using different *dcl* mutants but these do not exist in *N. benthamiana* at the moment.

### Functional analysis of vsiRNAs

It was shown before that vsiRNAs do mediate cleavage of viral RNAs [24]. We asked the question whether viral sequences complementary to abundant vsiRNAs (i.e.: generated from hot spots) are targeted more efficiently than sequences that are recognised by vsiRNAs generated from non-hot spot regions. Interestingly, the abundance of vsiRNAs complementary to the sensor constructs did not show any correlation with targeting efficiency of the different sensors. There are several possible explanation for this. First, if hot spot regions form partially double stranded structures with other regions of the viral RNA it would mean that these regions are less efficiently targeted by vsiRNAs since it was reported that RNAs with strong local structures are less accessible for RISC mediated cleavages [18],[29]. The other potential explanation is that primary vsiRNAs are not good effectors of gene silencing. Indeed only small portion of vsiRNAs were found in high molecular weight complexes [24]. Moreover a recent report demonstrated that vsiRNAs produced in the absence of RDR6 are poor effectors of gene silencing [30]. We showed that most CymRSV vsiRNAs are primary siRNAs including the ones derived from hot spots, although, it is possible that the vsiRNAs, which efficiently target the viral RNA are the small amount of secondary siRNAs (DCL-D or DCL-I) that may be generated in CymRSV infected plants. However, we do not have evidence for this latter possibility.

## Methods

### Bioinformatic analysis

Adaptors were removed from both 454 and Solexa samples by searching for the last eight bases of the 5′ adaptor (in the case of 454 samples) and the first eight bases of the 3′ adaptor (for both 454 and Solexa samples) using the UEA sRNA tools adaptor removal application [31] The sequence after the 5′ adaptor match and before the 3′ adaptor match was retained for further analysis.

vsiRNA sequences were mapped to the CymRSV genome using PatMaN [32] allowing a single mismatch and zero gaps. vsiRNA abundances in each sample were normalised by dividing each count by the total number of trimmed reads for a given sample and then multiplying this value by one million. This gave a value of counts per million reads and ensured that the profiles were comparable even though the sample sizes varied. Profiles were plotted for each sample by taking the sum of the normalised abundance for each vsiRNA sequence covering a given nucleotide position on the viral genome.

Boundaries of hot spot regions were initially determined using a simple algorithm designed to detect peaks in the profile then checked manually on the graphical plots. Sequence logos (Figure S7) were drawn for vsiRNA size classes of 20, 21, and 22 nucleotides, respectively, using the program seqlogo from the WebLogo package version 2.8.2 [33].

A Perl script was written to identify vsiRNAs that could potentially base-pair with each other. Firstly sRNA sequences from the CymRSV PHOS Solexa sample were mapped to the plus strand of the CymRSV genome using PatMaN, allowing for one mismatch to the reference genome. All sequences matching to the plus strand of the CymRSV genome were then given as input to the script which used FASTA3 [34] to search each vsiRNA against the reverse complement of all plus strand genome matching sequences. Any resulting matches were then aligned to the query sequence using ClustalW [35] before finding the complement of the hit sequence to obtain the correct orientation. Any potential duplexes with four or fewer mismatches including a maximum of one bulge were accepted as potential pairs. This process was then repeated separately for the sequences matching to the minus strand of the CymRSV genome.

### *Agrobacterium tumefaciens* infiltration, RNA isolation and hybridization analyses

*A. tumefaciens* infiltration was carried out according to Silhavy et al. (2002). For coinfiltration of *N. benthamiana* leaves, mixtures of strains carrying sensor constructs (OD_600_ = 0.15) and strains carrying suppressor constructs (OD_600_ = 0.3) were used. Total RNA from *Agrobacterium*-infiltrated *N. benthamiana* and virus infected plant leaves was isolated using Trizol reagent [36]. Denaturing RNA gel blot hybridisation and analyses were done as described previously [37].

### Preparation of sensor constructs

All sensor constructs were prepared from the previously reported 35S-green fluorescent protein (GFP) binary plasmid [38]. First, a SmaI restriction site was inserted downstream of the GFP ORF by using the QuikChange site-directed mutagenesis kit (Stratagene) by following the instruction manual. The PCR fragment of 200 bp, corresponding to the indicated positions (Figure 5) of the CymRSV genome (accession no. NC 003532), was amplified by using appropriate 5′-phosphorylated oligonucleotides and placed into the unique SmaI site of the modified 35S-GFP plasmid. The plus and minus orientations of the inserts were selected, thus generating the different sensor constructs shown in Figure 5.

### *In vitro* RNA transcription and plant inoculation

*In vitro* transcription of CymRSV and Cym19stop RNAs from linearized template plasmids and inoculation of RNA transcripts onto *Nicotiana benthamiana*, *N. benthamiana* GFP16c/RDR6i line [15] and *N. clevelandii* plants were performed as described previously [39].

### RNA extraction from virus infected plants

Total RNA was extracted from 100 mg of systemicly infected leaf tissue [40]. Briefly, the homogenized plant materials were resuspended in 600 µL of extraction buffer (0.1 M glycine-NaOH, pH 9.0, 100 mM NaCl, 10 mM EDTA, 2% SDS, and 1% sodium lauroylsarcosine) and mixed with an equal volume of phenol. The aqueous phase was treated with equal volumes of phenol and chloroform, precipitated with ethanol, and resuspended in sterile water and used in subsequent reactions.

### 454 sequencing of small RNAs

Small RNA between 19–24nt were cloned from systemic leaves of *N. benthamiana* as described by Moxon et al. [41]. Briefly, the sRNA fraction was purified and ligated to adaptors without de-phosphorylating and re- phosphorylating the sRNA. The RNA was reverse transcribed, and amplified by PCR before being sent to 454 Life Sciences for pyrosequencing. The small RNA libraries are submitted to GEO and can be accessed through a super series number: GSE17278.

### Solexa sequencing of small RNAs

Small RNA fraction (19–24 nt) of total RNA extracted from systemic leaves of *N. benthamiana* was isolated from 15% denaturing polyacrylamide gel. The eluted sRNA fraction was divided into two and one half was not treated (called CymRSV-PHOS and Cym19stop-PHOS) while the other half was de-phosphorylated with Shrimp Alkaline Phosphatase and re- phosphorylated with T4 Polynucleotide Kinase (called CymRSV-CIP and Cym19stop-CIP). The resulting sRNAs were ligated to adaptors in the following reaction. The purified, adaptor ligated short RNAs were converted to DNA by RT-PCR and the DNA was sequenced on a Solexa platform (Illumina). The small RNA libraries are submitted to GEO and can be accessed through a super series number: GSE17278.

### Isolation, labeling and hybridization of small RNAs from virus infected plants

For preparation of 19–24 nt RNA fraction, 15 to 20 µg of total RNA from *in vitro* transcribed virus infected plants was subjected to electrophoresis through an 8% denaturing polyacrylamide gel followed by staining in 1× Tris-borate-EDTA and 0.5 µg/mL ethidium bromide solutions for 5 min. The 19–24 nt fraction was visualized by UV light and excised from the gel. The gel slice was crushed, covered with 2 volumes of elution buffer (80% formamide, 40 mM Pipes, pH 6.4, 1 mM EDTA, and 400 mM NaCl) and incubated overnight. The gel residues were pelleted by centrifugation and the supernatant was precipitated with ethanol. The RNA (∼100 ng) was dephosphorylated and then labeled in a 10-µl reaction in the presence of γ-^32^P-ATP and RNasin with 8 units of T4 polynucleotide kinase. The labeled 19–24 nt RNAs were used for hybridization either to membranes (Zeta-Probe GT, BioRad) containing five hundred 21mer DNA oligonucleotides in a one nucleotide sliding window covering a 520 nt region (nucleotides 2650–3169 on the positive strand, see oligonucleotide sequences in Table S3) or to a membrane containing hundred eighty-six 21mer DNA oligonucleotides in a one nucleotide sliding window covering a 206 nt region (nucleotides 4300–4505 on the positive strand, see oligonucleotide sequences in Table S3). As a control for cross hybridization two set of oligonucleotides, which were one to five nucleotides shorter at their 5′ ends were also spotted on the membranes (12-1, 12-2, 12-3, 12-4, 12-5, 87-1, 87-2, 87-3, 87-4 and 87-5). As a negative control to check background hybridization we spotted pUC/M13 Forward and Reverse primer onto the membranes or only water. Hybridization was performed in Ambion ULTRAhyb-Oligo Buffer at 37°C following the manifacturer's instruction. Signals were quantified with a Genius Image Analyzer (Syngene).

### Terminator digest

19mer and 21 mer synthetic RNA molecules were phosphorylated and 30 µl of sense RNA (10 µM) and 30 µl of antisense RNA (10 µM) were incubated in 90 µl annealing buffer (30 mM HEPES-KOH pH 7.4, 100mM KCl, 2mM MgCl2, 50mM ammonium acetate) to form siRNA duplex (19 nt siRNA, siRNA171a and siRNA171b). We also prepared mismatch containing siRNA duplexes on the same way by incubating sense RNA with different mismatch containing antisense RNA (19mer antisense RNA 1mism_end, 19mer antisense RNA 2mism_end, 19mer antisense RNA 3mism_end, 19mer antisense RNA 3mism_middl, 2mm antisense siRNA171a and 3mm antisense siRNA171c). We digested the resulting siRNA duplexes for one hour at 30°C in decreasing concentration (2 µM, 0,4 µM, 0,2 µM and 0,04 µM or 0,1 µM, 0,02 µM, 0,004 µM and 0,002 µM and as a control 2 µM or 0,002 µM sense phosphorylated RNA oligo) in a 10 µl reaction in the presence of 0.25 U Terminator™ 5′-Phosphate-Dependent Exonuclease (Epicentre Biotechnologies) without or after 1 minute 90°C denaturation. Terminator treated and non-treated samples were loaded onto 0.5× TBE 15% UREA-Polyacrylamide gel. After electrophoresis the samples were transferred to Zeta-probe GT BioRad membranes by Semi-dry blotting. The membranes were hybridized with γ-^32^P-ATP labelled DNA oligonucleotide (19mer sense detector, siRNA171a sense detector and siRNA171b detector) complementary to the 19mer sense RNA or to the sense RNE of siRNA171a or to the sense RNA of siRNA171b respectively. Hybridization was performed in Ambion ULTRAhyb-Oligo Buffer at 37°C following the manifacturer′s instruction.

RNA extracted from CymRSV infected plants was mixed with 19mer siRNA duplex and was diluted containing decreasing concentration of total RNA and siRNA duplex (2,3 µg RNA+2 µM siRNA duplex, 0,46 µg RNA+0,4 µM siRNA duplex, 0,23 µg RNA+0,2 µM siRNA duplex, 0.046 µg RNA+0,04 µM siRNA duplex, and as a control 2,3 µg RNA+2 µM sense phosphorylated RNA oligo). We digested the resulting RNA and siRNA duplex mixture in a 10 µl reaction in the presence of 0.25 U Terminator™ 5′-Phosphate-Dependent Exonuclease without or after 1 minute 90°C denaturation for one hour at 30°C. Terminator treated and non-treated samples were loaded onto 0.5× TBE 15% UREA-Polyacrylamide gel. After electrophoresis the samples were transferred to Zeta-probe GT BioRad membranes by Semi-dry blotting. The membranes were hybridized with γ-^32^P-ATP labelled DNA oligonucleotides. Cym mix probe was a pool of five oligonucleotides (cym3125, cym3196, cym3420, cym3950 and cym4240) and complenetary to the negative strand of the virus genome. Cym3025 was also complementary to the negative strand of the virus genome. Cym minus probe was a pool of two oligonucleotides (cym1021 minus and cym3710 minus) and complementary to the positive strand of the virus genome. Cym 4632 LNA was also complementary to the positive strand of the virus genome. U6 detects the U6 snRNA while miR159 LNA detects the mature strand of miR159 and contains locked nucleic acid (LNA) nucleotides. The oligonucleotide sequences used for hybridisation are available in Table S3. Hybridization was performed in Ambion ULTRAhyb-Oligo Buffer at 37°C following the manifacturer's instruction.

## Supporting Information

Figure S1 454 profile of CymRSV and Cym19stop vsiRNAs. The normalised number of vsiRNAs in the two 454 datasets (A: wild type virus; B: 19stop mutant) containing each nucleotide in the virus genome was plotted against the positions of nucleotides in the viral genome.
(10.19 MB TIF)

Figure S2 Terminator digest of + strand vsiRNAs. The membranes shown on Figure 2 were re-probed with a locked nucleic acid (LNA) probe that detects a + strand vsiRNA at the position 4632–4653. In addition, two new membranes were prepared where the samples were separated much better (longer run). These membranes were hybridized with a Cym minus probe, which was a pool of two oligonucleotides (cym1021 minus and cym3710 minus), both complementary to the positive strand of the virus genome. The 21- and 22-mer vsiRNA species are well separated on these membranes.
(0.31 MB JPG)

Figure S3 Terminator digest of perfect and imperfect duplex synthetic siRNAs. The terminator assay was carried out as described for Figures 2 and 3. Briefly: *in vitro* synthesised and phosphorylated siRNAs were annealed to each other to generate either perfect duplexes or imperfect duplexes (A). Please note that the imperfect duplexes contain mismatches and additional U∶G pairs. The perfect duplexes were digested with Terminator™ 5′-Phosphate-Dependent Exonuclease in decreasing concentration without (left panel) or after denaturation (right panel) (B). The two imperfect duplexes were also digested with Terminator™ 5′-Phosphate-Dependent Exonuclease in decreasing concentration without denaturation (C). The efficiency of the digestion was monitored by northern blot assay using a probe complementary to one of the strands of the synthetic siRNAs.
(0.21 MB JPG)

Figure S4 Accumulation of genomic viral RNA in wild type and p19 mutant virus infected plants. Total RNA was extracted from wild type and p19 mutant virus infected plants. The RNA was separated on 1.2% denaturing formaldehyde agarose gels and blotted to membranes. The membranes were hybridised with + strand, − strand or actin specific probes. All four samples contained less + strand RNA and more − strand (especially subgenomic RNA 2) in the mutant virus infected plants. G: genomic viral RNA; sg1: subgenomic RNA 1; sg2: subgenomic RNA 2.
(0.07 MB JPG)

Figure S5 Simulation of the effect of sequencing errors on positive and negative strand vsiRNAs. The effect of sequencing errors on positive and negative strand vsiRNAs was simulated by applying sequencing errors at different rates (1 error in 1000, 100 or 10 sequences) on perfectly matching positive and negative strand vsiRNAs (either redundant or non-redundant sequences). The ratio of perfectly matching sequences to those matching with 0 or 1 mismatch was calculated. We found a similar effect on the positive and negative strand vsiRNAs when redundant reads were used but a much more pronounced effect on the positive strand vsiRNAs when the simulation was applied to non-redundant reads.
(0.03 MB XLS)

Figure S6 Comparison of 454 and Solexa profiles of vsiRNAs. Profiles of vsiRNA obtained by the 454 and Solexa platforms are shown on the top of each other. Red and blue lines represent 454 and Solexa profiles, respectively.
(0.12 MB JPG)

Figure S7 Nucleotide distributions in vsiRNAs 5′ end. First nucleotides of vsiRNAs obtained through Solexa sequencing of the four small RNA libraries were analysed for each size category (18–24 nucleotides). The top four and bottom panels show the result for all reads and hot spots, respectively.
(0.32 MB JPG)

Figure S8 Separate Solexa profiles of 21 and 22 nt vsiRNAs. The 21 and 22 nt vsiRNAs were separated from the Solexa datasets (wild type and mutant virus), and their normalised number was plotted against the positions of nucleotides in the viral genome. Short RNAs were sequenced from wild type (G11) or silencing protein disabled (19) virus infected *N. benthamiana* following two different protocols. The 5′ adapter was either directly ligated to the short RNAs (PHOS) or first depohosphorylated and then re-phosphorylated before adapter ligation (CIP).
(0.16 MB JPG)

Table S1 Potential vsiRNA duplexes derived from the + strand. All sequences matching to the plus strand of the CymRSV genome were searched against the reverse complement of all plus strand genome matching sequences. Potential duplexes with four or fewer mismatches and a maximum of one bulge are listed. The number of reads is shown in brackets after each sequence.
(1.29 MB TXT)

Table S2 Potential vsiRNA duplexes derived from the − strand. All sequences matching to the minus strand of the CymRSV genome were searched against the reverse complement of all minus strand genome matching sequences. Potential duplexes with four or fewer mismatches and a maximum of one bulge are listed. The number of reads is shown in brackets after each sequence.
(0.56 MB TXT)

Table S3 Sequences of oligonucleotides. Sequences of oligonucleotides used for the dot blot and northern blots are listed.
(0.09 MB XLS)
