# Supplementary figures and images for: Structural and Functional Analysis of Viral siRNAs
Source: PLoS Pathog. 2010 Apr 1;6(4):e1000838. doi: 10.1371/journal.ppat.1000838 (PMC2848561; doi:10.1371/journal.ppat.1000838)

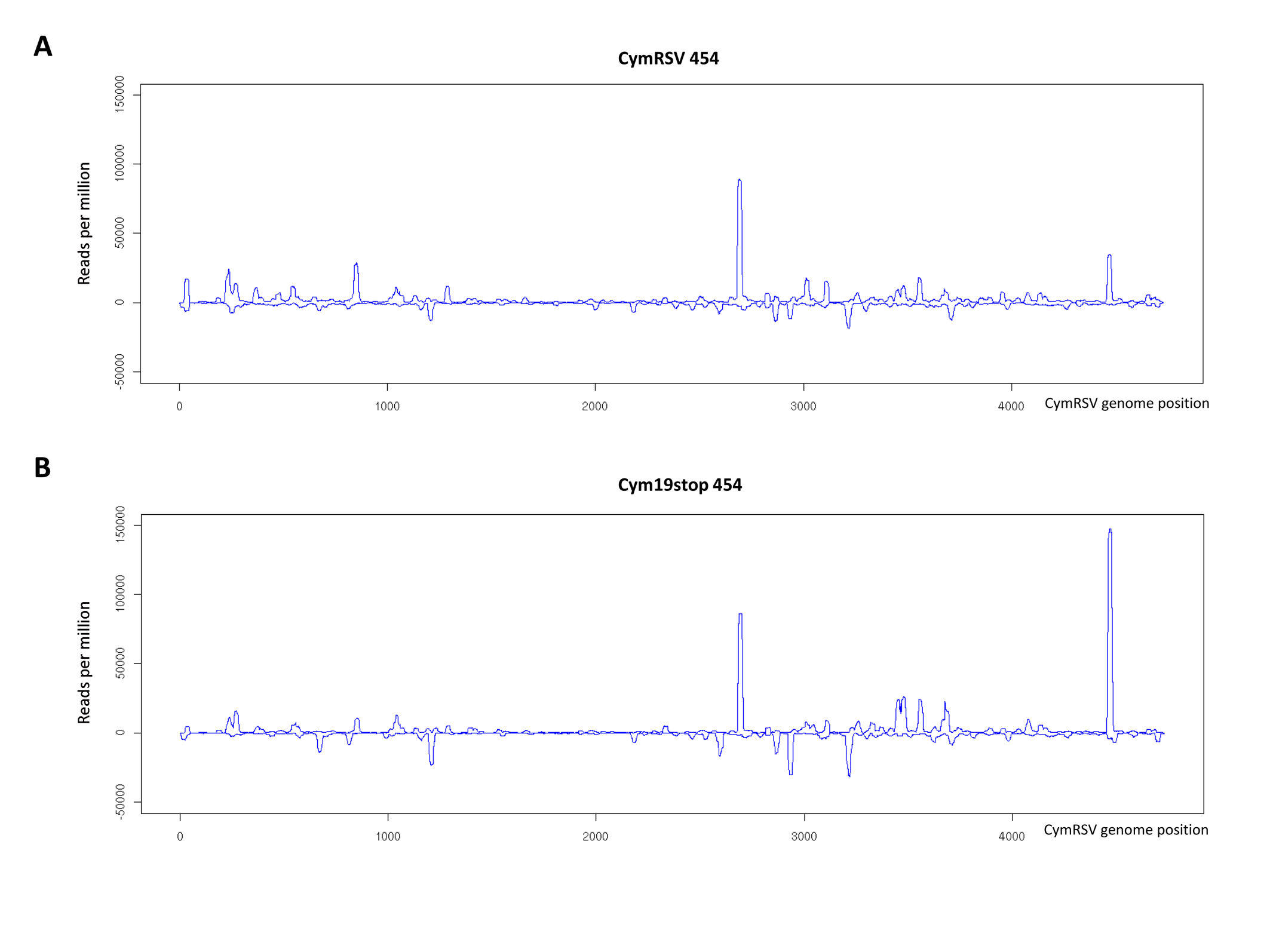

Supplement: Figure S1 — 454 profile of CymRSV and Cym19stop vsiRNAs. The normalised number of vsiRNAs in the two 454 datasets (A: wild type virus; B: 19stop mutant) containing each nucleotide in the virus genome was plotted against the positions of nucleotides in the viral genome. (10.19 MB TIF) [file ppat.1000838.s001.tif]

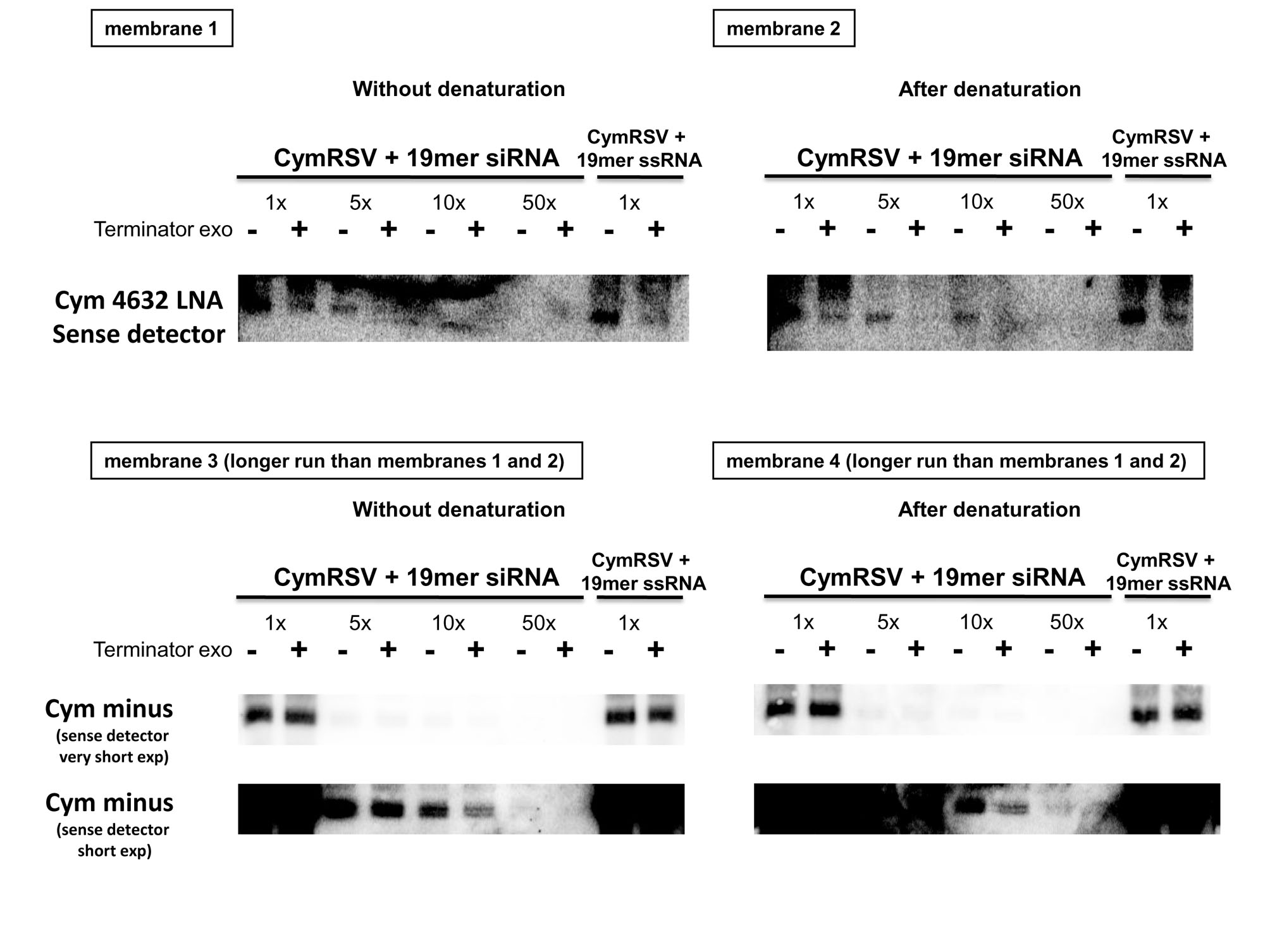

Supplement: Figure S2 — Terminator digest of + strand vsiRNAs. The membranes shown on Figure 2 were re-probed with a locked nucleic acid (LNA) probe that detects a + strand vsiRNA at the position 4632–4653. In addition, two new membranes were prepared where the samples were separated much better (longer run). These membranes were hybridized with a Cym minus probe, which was a pool of two oligonucleotides (cym1021 minus and cym3710 minus), both complementary to the positive strand of the virus genome. The 21- and 22-mer vsiRNA species are well separated on these membranes. (0.31 MB JPG) [file ppat.1000838.s002.jpg]

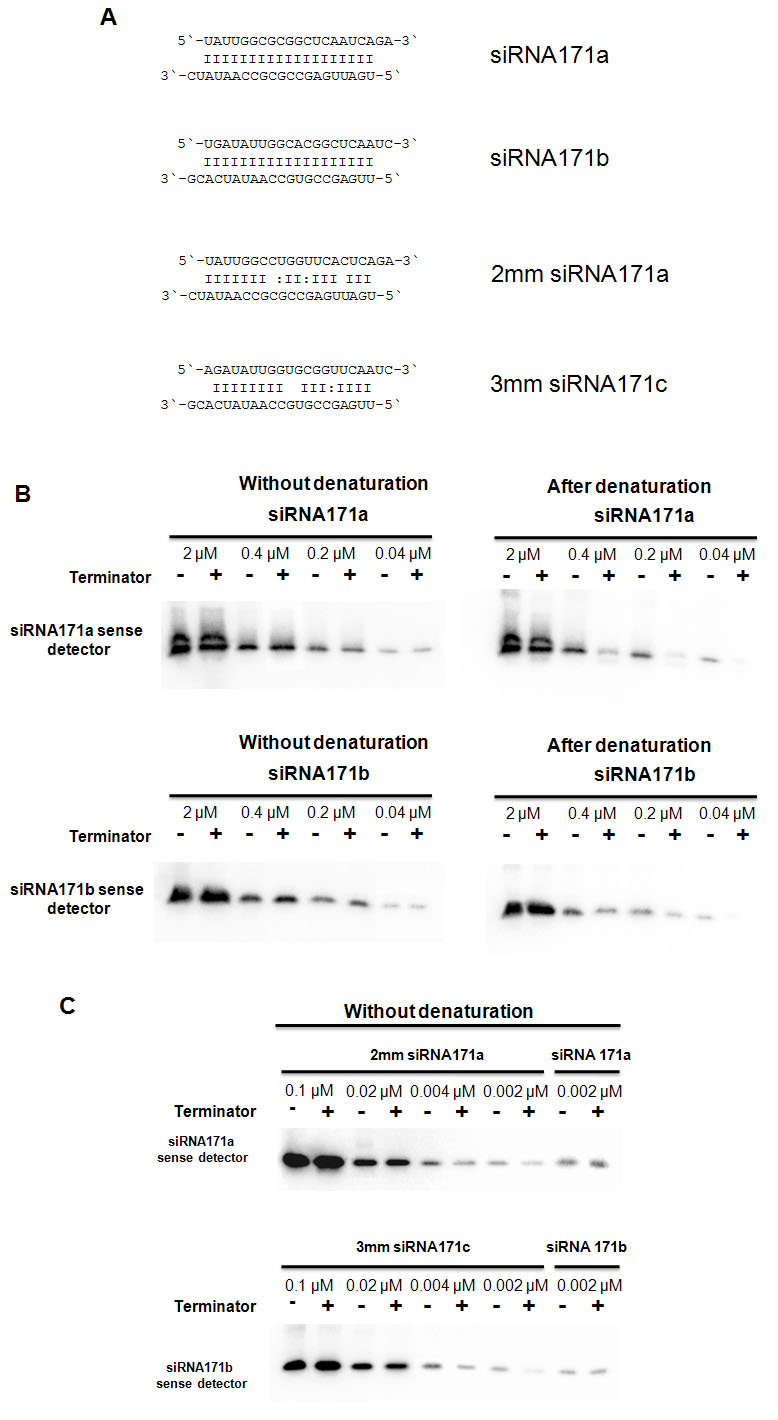

Supplement: Figure S3 — Terminator digest of perfect and imperfect duplex synthetic siRNAs. The terminator assay was carried out as described for Figures 2 and 3. Briefly: in vitro synthesised and phosphorylated siRNAs were annealed to each other to generate either perfect duplexes or imperfect duplexes (A). Please note that the imperfect duplexes contain mismatches and additional U∶G pairs. The perfect duplexes were digested with Terminator™ 5′-Phosphate-Dependent Exonuclease in decreasing concentration without (left panel) or after denaturation (right panel) (B). The two imperfect duplexes were also digested with Terminator™ 5′-Phosphate-Dependent Exonuclease in decreasing concentration without denaturation (C). The efficiency of the digestion was monitored by northern blot assay using a probe complementary to one of the strands of the synthetic siRNAs. (0.21 MB JPG) [file ppat.1000838.s003.jpg]

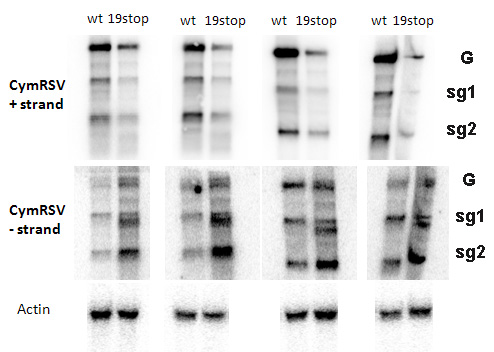

Supplement: Figure S4 — Accumulation of genomic viral RNA in wild type and p19 mutant virus infected plants. Total RNA was extracted from wild type and p19 mutant virus infected plants. The RNA was separated on 1.2% denaturing formaldehyde agarose gels and blotted to membranes. The membranes were hybridised with + strand, − strand or actin specific probes. All four samples contained less + strand RNA and more − strand (especially subgenomic RNA 2) in the mutant virus infected plants. G: genomic viral RNA; sg1: subgenomic RNA 1; sg2: subgenomic RNA 2. (0.07 MB JPG) [file ppat.1000838.s004.jpg]

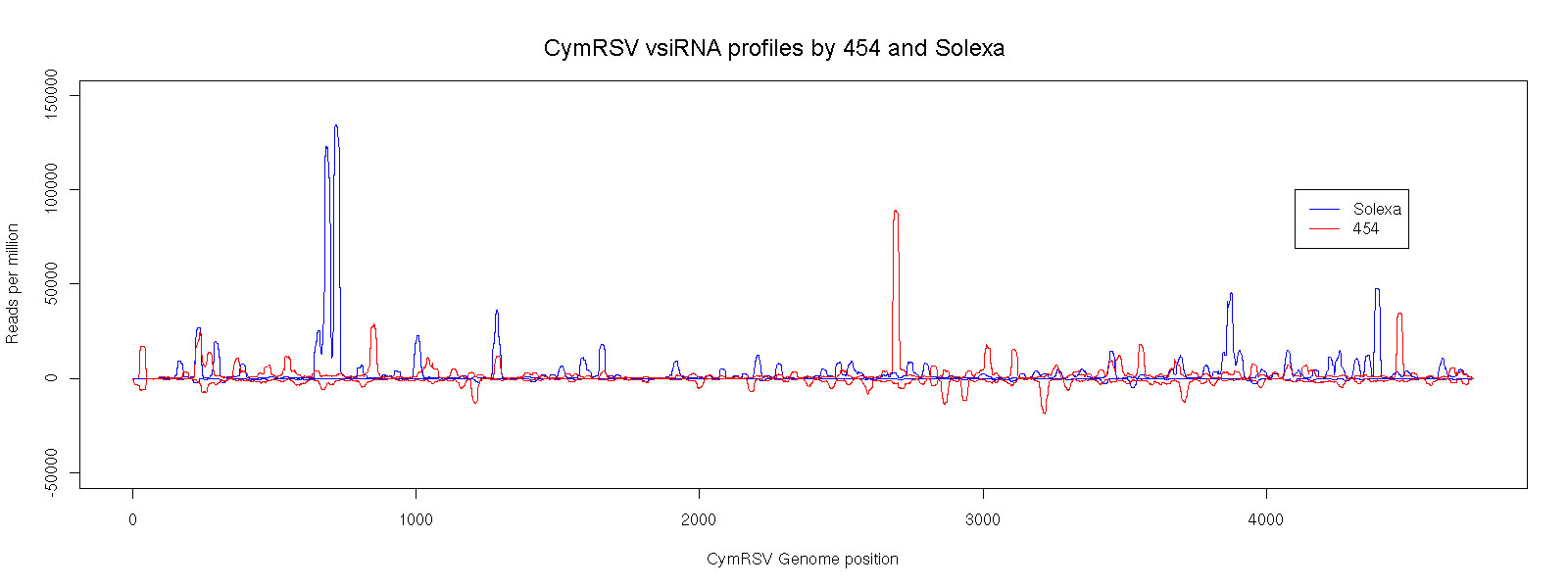

Supplement: Figure S6 — Comparison of 454 and Solexa profiles of vsiRNAs. Profiles of vsiRNA obtained by the 454 and Solexa platforms are shown on the top of each other. Red and blue lines represent 454 and Solexa profiles, respectively. (0.12 MB JPG) [file ppat.1000838.s006.jpg]

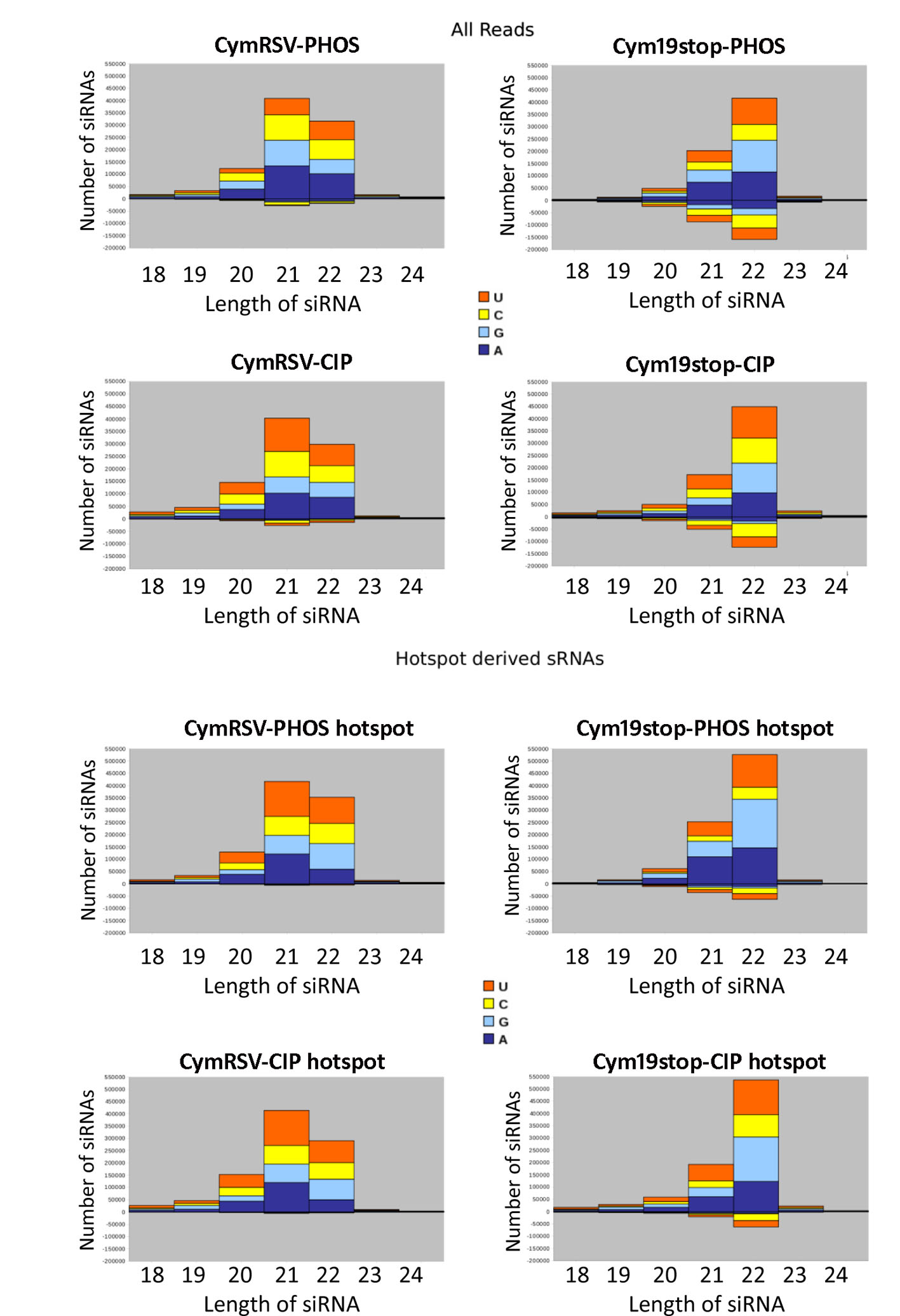

Supplement: Figure S7 — Nucleotide distributions in vsiRNAs 5′ end. First nucleotides of vsiRNAs obtained through Solexa sequencing of the four small RNA libraries were analysed for each size category (18–24 nucleotides). The top four and bottom panels show the result for all reads and hot spots, respectively. (0.32 MB JPG) [file ppat.1000838.s007.jpg]

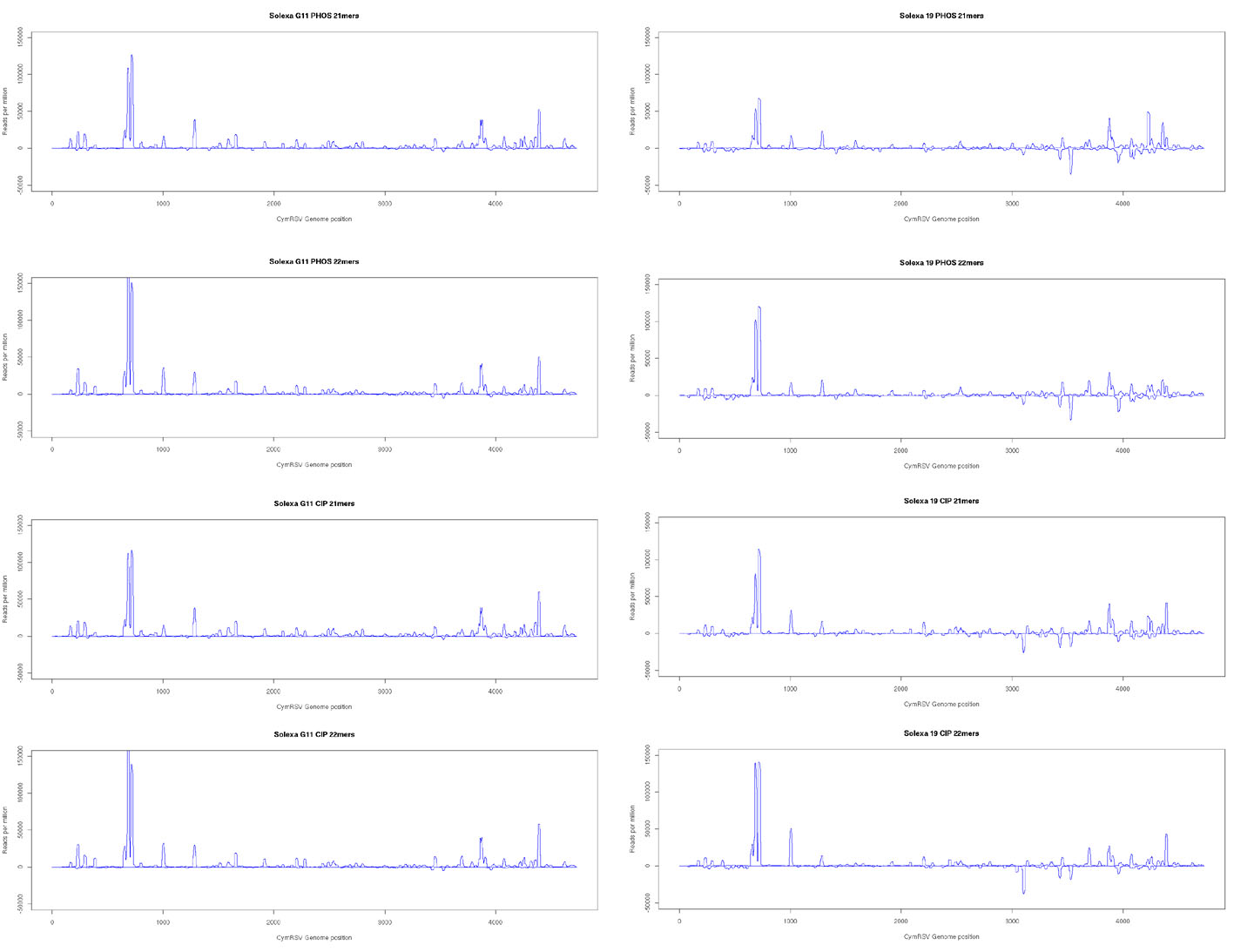

Supplement: Figure S8 — Separate Solexa profiles of 21 and 22 nt vsiRNAs. The 21 and 22 nt vsiRNAs were separated from the Solexa datasets (wild type and mutant virus), and their normalised number was plotted against the positions of nucleotides in the viral genome. Short RNAs were sequenced from wild type (G11) or silencing protein disabled (19) virus infected N. benthamiana following two different protocols. The 5′ adapter was either directly ligated to the short RNAs (PHOS) or first depohosphorylated and then re-phosphorylated before adapter ligation (CIP). (0.16 MB JPG) [file ppat.1000838.s008.jpg]
